# Dr. Guilford W. McCray

**Published:** 1904-05

**Authors:** 


### OBITUARY NOTES.

Dr. Guilford W. McCray, of Buffalo, died of apoplexy, at his residence, April 18, 1904, aged 73 years. He took his medical degree at Castleton, Vt., in 1857. He practised first at Varysburg and afterward at Cowlesville, N. Y. He came to Buffalo in 1858, and associated himself with the late Dr. S. L. Grosvenor in the drug business for many years. After the latter's death, Dr. McCray continued the business independently for a number of years in the retail line, but for the past twenty years he conducted a wholesale drug establishment.

